# Dietary intake patterns and KDOQI guideline compliance among hemodialysis patients in Bangladesh: Gaps, misreporting, and clinical implications

**DOI:** 10.1371/journal.pone.0360654

**Published:** 2026-10-12

**Authors:** Farhana Akter, Tanjina Rahman, Shakil Ahmed, Md Sajjadul Haque Ripon, Marium Sultana, Nasrin Sultana, Harun-ur Rashid

**Affiliations:** 1 Department of Food Technology and Nutrition Science, Noakhali Science and Technology University, Noakhali, Bangladesh; 2 Institute of Nutrition and Food Science, University of Dhaka, Dhaka, Bangladesh; 3 Department of Nutrition and Food Engineering, Daffodil International University, Dhaka, Bangladesh; 4 Department of Nephrology, Kidney Foundation Hospital and Research Institute, Dhaka, Bangladesh; Kidney and Urology Center, EGYPT

## Abstract

Optimal nutrition is a central to the care of people receiving maintenance hemodialysis, yet dietary intake in these patients is usually measured by self-report, and self-report is known to be unreliable. Evidence from resource-limited dialysis settings is scarce. This cross-sectional study assessed dietary intake among 181 adults receiving maintenance hemodialysis at four hospital-based units in Bangladesh, and examined how screening reported intake for plausibility changes conclusions about guideline adherence. Intake was measured by three non-consecutive 24-hour recalls and compared with the Kidney Disease Outcomes Quality Initiative (KDOQI) recommendations. Reporting plausibility was classified from the ratio of energy intake to estimated basal metabolic rate. Appetite was assessed using the Appetite and Diet Assessment Tool. Of 178 participants with complete data, 65.2% reported acceptable, 32.0% under-reported and 2.8% over-reported energy intake. In the cohort as a whole, only 20 of 176 (11.4%) met the energy target of 30–35 kcal/kg/day and 58 (33.0%) met the protein target of at least 1.0 g/kg/day. Screening changed these estimates in opposite directions according to the type of target. For intake targets, adherence rose once implausible reports were excluded: energy from 11.4% to 15.5% and protein from 33.0% to 47.4%. For restriction targets the reverse occurred: apparent adherence to the sodium limit fell from 73.6% to 65.5% and to the phosphorus limit from 88.2% to 84.5%. The phosphorus-to-protein ratio exceeded 12 mg/g in all but five participants. Poor appetite was common (42.0%) but did not correspond to lower measured intake, and was instead associated with reporting plausibility (p = 0.024). Unscreened self-reported intake simultaneously overstates dietary inadequacy and understates dietary excess, and should not be used unfiltered to guide nutritional decisions in this population.

## Introduction

Chronic kidney disease (CKD) is a growing global public health challenge, affecting an estimated 8–16% of adults worldwide [1], and characterized by the progressive loss of kidney function over time [2]. In 2017, approximately 850 million people were living with CKD [3]. The burden is disproportionately high in low- and middle-income countries (LMICs), including Bangladesh, where CKD prevalence ranges from 17.3% to 22.4% [4]. Each year, 35,000–40,000 Bangladeshis progress to end-stage renal disease (ESRD), and nearly 40,000 die due to CKD-related complications [5]. Once ESRD develops, renal replacement therapy, usually hemodialysis in resource-limited settings becomes essential for survival [6].

Nutrition plays a central role in preserving residual kidney function and improving clinical outcomes among CKD and hemodialysis patients [7]. Yet, inadequate dietary intake is common during maintenance dialysis, and patients frequently fail to meet nutrient requirements outlined by the NKF/KDOQI guidelines [8]. These guidelines specify individualized targets for macro- and micronutrient consumption to reduce risks of hyperphosphatemia, hyperkalaemia, hypertension, and fluid overload [9]. Evidence suggests that higher-quality diets may lower the risk of ESRD progression and mortality among patients with CKD stages 3–5 [10]. However, many patients have limited knowledge of renal-specific dietary recommendations, leading to poor adherence and suboptimal nutritional status [11]. Adequate nutritional management is therefore essential to maintain serum biochemical balance and prevent protein-energy wasting [12].

Accurate dietary assessment is equally critical. Misreporting of food intake, particularly underreporting can distort clinical interpretation and lead to inappropriate dietary counselling, exacerbating complications such as hyperkalaemia, fluid overload, malnutrition, and mineral-bone disorders [13]. Misreporting remains a persistent challenge in hemodialysis research and practice [14], with higher prevalence among women, younger individuals, and those with elevated body weight [15]. Biomarkers such as the energy intake–basal metabolic rate ratio (EI:BMR) are commonly used to detect implausible intake reports and strengthen data validity [16].

Appetite is another key determinant of dietary intake in hemodialysis patients. Reduced appetite is widely reported across diverse populations with kidney disease and contributes to protein-energy malnutrition, affecting 30–75% of hemodialysis patients [17,18]. Appetite assessment tools, including the single-item Appetite and Diet Assessment Tool (ADAT), offer a simple yet informative measure for identifying patients at nutritional risk [19].

Despite the high and rising burden of CKD in Bangladesh, evidence on dietary intake, misreporting patterns, appetite status, and adherence to KDOQI recommendations among hemodialysis patients remains scarce. Understanding these factors is particularly important in resource-poor settings, where dietary counselling services, nutrition education, and trained renal dietitians are limited.

This study therefore had two aims. The primary aim was to determine how screening self-reported dietary intake for plausibility alters estimates of adherence to KDOQI energy, protein and mineral targets in a Bangladeshi hemodialysis cohort. The secondary aims were to quantify macronutrient and micronutrient intake using a three-day 24-hour recall analysed with locally adapted food composition data, to identify independent predictors of under-reporting, and to examine whether self-rated appetite corresponds to measured intake. Together, these objectives were designed to generate context-specific evidence that can guide more accurate nutritional assessment and inform targeted dietary interventions in resource-limited hemodialysis settings.

## Methodology

### Study design and population

A descriptive cross-sectional study was conducted across four districts in Bangladesh (Dhaka, Cumilla, Noakhali, and Mymensingh). A total of 181 adult patients undergoing maintenance hemodialysis were recruited from four hospital-based dialysis units, one in each study district. Participants were enrolled by consecutive convenience sampling: every patient attending a scheduled dialysis session during the recruitment window who met the eligibility criteria was invited to take part, and no stratification or randomisation by centre was applied.

Eligibility criteria were applied as follows. Inclusion criteria were: (i) age ≥ 18 years; (ii) receipt of maintenance hemodialysis for at least three months; (iii) exclusively oral food intake, with or without oral nutritional supplements; (iv) clinically stable condition, defined as no hospitalisation or acute intercurrent illness in the preceding two weeks; and (v) ability and willingness to provide written informed consent. Exclusion criteria were: (i) enteral tube feeding or parenteral nutrition; (ii) acute kidney injury superimposed on CKD, active infection or sepsis at the time of assessment; (iii) cognitive, hearing, or language impairment precluding reliable completion of a 24-hour dietary recall; (iv) pregnancy or lactation; and (v) incomplete dietary or anthropometric records required for the calculation of reporting validity.

No formal a priori power calculation was performed, because the study was designed to describe dietary intake patterns and guideline adherence rather than to test a pre-specified hypothesis. The achieved sample of 181 participants was determined by the number of eligible patients attending the participating units during the six-month recruitment period. For a descriptive proportion of approximately 50%, this sample size yields a two-sided 95% confidence interval of about ±7.3 percentage points, which was considered adequate for the descriptive objectives of the study.

### Ethical considerations

Data were collected between January 2022 and June 2022 using a pre-tested questionnaire administered during HD sessions. All participants provided written informed consent prior to data collection. Ethical approval was granted by the Research Cell Committee of Noakhali Science and Technology University (NSTU/RC-FTNS/MS/21/72). Permissions were also obtained from all participating dialysis centers in the four study districts.

### Dietary assessment

Dietary intake was evaluated using a validated three-day 24-hour dietary recall (3DDR), which included one dialysis day, one non-dialysis day, and one weekend day. All recalls were administered face-to-face at the dialysis unit by nutrition graduates of the Department of Food Technology and Nutrition Science, Noakhali Science and Technology University, who were trained in the multiple-pass 24-hour recall method and supervised by the study nutritionists; a standardised probing script was used to reduce interviewer variability. Standardized household measuring tools and an electronic kitchen scale were used to estimate portion sizes. Collected food items and recipes were entered into Nutritionist Pro™ (Axxya Systems LLC, Stafford, TX, USA) using food composition data from the *Food Composition Table for Bangladesh* [20]. Approximately 381 food items and 150 mixed Bangladeshi recipes were incorporated. Nutrient intakes, including energy, macronutrients, and selected micronutrients were quantified and compared against current NKF/KDOQI nutrition recommendations for maintenance dialysis [7,21].

#### Reference standards and definition of adherence.

Dietary adequacy was benchmarked against the KDOQI clinical practice guideline for nutrition in CKD [7,21]. Energy adequacy was defined as a dietary energy intake (DEI) of 30–35 kcal/kg of post-dialysis body weight per day. For protein, the 2020 KDOQI update recommends a dietary protein intake (DPI) of 1.0–1.2 g/kg/day for metabolically stable patients on maintenance hemodialysis [21]; we therefore report adherence against both bounds of this range, using ≥1.2 g/kg/day as the primary (upper-bound) target for comparability with earlier literature and ≥1.0 g/kg/day as a secondary (minimum acceptable) target.

Throughout this paper, ‘compliance’ refers to adherence to a single, explicitly named nutrient target and not to a composite score. Specifically, energy compliance denotes the proportion of patients with DEI of 30–35 kcal/kg/day, and protein compliance denotes the proportion with DPI at or above the stated threshold. Composite adherence, defined as meeting the energy and protein targets simultaneously, is reported separately and is identified as such wherever it appears. Weight-adjusted intake data were available for 176 of the 178 participants in the analytic sample, and this is the denominator for every adherence figure reported.

### Assessment of Reporting Plausibility

To identify misreporting, the ratio of energy intake to basal metabolic rate (EI:BMR) was calculated. The ratio was adjusted for an assumed physical activity level (PAL) of 1.2 reflecting the characteristically sedentary lifestyle of maintenance hemodialysis patients and consistent with a comparable cut-off applied in this same clinical population [22]. This PAL value also corresponds to the lower bound of physiologically plausible activity described in doubly-labelled-water studies of chair- or bed-bound individuals [23]. Cut-off values of <0.8, 0.8–1.3, and >1.3 were used to classify under reporters, acceptable reporters, and over reporters, respectively [23,24]. These thresholds correspond to the Goldberg cut-offs, which are derived from the physiological limits of sustainable energy balance and have been applied to dialysis and CKD cohorts in previous work, including studies of underreporting in maintenance hemodialysis [25–27]. Because the Goldberg approach assumes weight stability, and because interdialytic fluid gain inflates pre-dialysis weight, post-dialysis (dry) body weight was used for all BMR and weight-adjusted intake calculations in this study. BMR was estimated using the Harris–Benedict equation for men [66.5 + (13.75 × weight in kg) + (5.0 × height in cm) – (6.76 × age in years)] and women [655 + (9.6 × weight in kg) + (1.8 × height in cm) – (4.7 × age in years)] [28].

### Anthropometric measurements

Height and weight (pre- and post-dialysis) were extracted from patient records. BMI was calculated as post-dialysis weight (kg)/height (m^2^) and categorized according to WHO standards [29]. Mid-arm circumference (MAC) was measured using a non-stretchable Lufkin® tape (Apex Tool Group, NC, USA), and triceps skinfold thickness (TSF) was assessed with Lange calipers (Power Systems, TN, USA). Mid-arm muscle circumference (MAMC) was computed using the formula:MAMC = MAC – (3.14 × TSF) [30].

### Biochemical parameters

Biochemical data were obtained from hospital case files and included serum albumin, total protein, creatinine, urea, sodium, potassium, calcium, phosphorus, and lipid profile components (total cholesterol and triglycerides).

### Appetite assessment

Appetite was assessed using the validated single-item Appetite and Diet Assessment Tool (ADAT). Participants rated their appetite over the previous week on a 5-point scale (1 = very good to 5 = very poor). For analysis, responses were grouped into “good” (very good/good), “fair,” and “poor” (poor/very poor). Lower ADAT scores indicated better appetite [17].

### Statistical analysis

Descriptive statistics were reported as frequencies (percentages) or mean ± standard deviation. Group differences were analyzed using one-way ANOVA for continuous variables and Pearson’s χ^2^ test for categorical variables. Because only five participants were classified as over reporters, several cross-tabulations contained expected cell counts below five; in these instances, Fisher’s exact test was used in place of Pearson’s χ^2^, and the test applied is indicated in the corresponding table footnote. To reduce the influence of this very small subgroup, a pre-specified sensitivity analysis was also performed in which under- and over reporters were combined into a single ‘invalid reporter’ category (n = 62) and compared with valid reporters (n = 116) using Welch’s independent-samples t-tests for continuous variables and χ^2^ or Fisher’s exact tests for categorical variables. Given the descriptive nature of the study and the number of nutrients examined, no formal adjustment for multiple comparisons was applied; nominal p-values are therefore reported, and isolated significant findings should be regarded as hypothesis-generating rather than confirmatory. Statistical significance was set at P < 0.05. Analyses were performed using IBM SPSS Statistics version 19.0 (IBM SPSS Inc., Chicago, IL, USA) and independently verified in Python 3.11 using the pandas, SciPy and stats models libraries.

## Results

### Demographic characteristics

Demographic findings are summarized in Tables 1 and 2. The study included 181 hemodialysis patients, comprising 96 males (53%) and 85 females (47%), with a mean age of 48 ± 13.8 years. Age distribution indicated 19.3% young adults, 47% middle-aged, and 33.7% older adults, with no gender-based differences. Most patients (80.1%) received dialysis twice weekly, with a mean session duration of 3.8 ± 0.2 hours. Dialysis vintage differed significantly by sex (40.6 ± 25.9 vs. 32.4 ± 23.7 months).

**Table 1:** Demographic and clinical characteristics of hemodialysis patients (n = 181).

Parameter	Total (n = 181)	Male (n = 96)	Female (n = 85)	*p*-Value
**Age (year**)	48 ± 13.8	48 ± 14.5	48.5 ± 13.1	0.837^a^
**Age category**				0.307^b^
Young	35 (19.3)	21 (21.9)	14 (16.5)	
Middle Aged	85 (47)	40 (41.7)	45 (52.9)	
Old	61 (33.7)	35 (36.5)	26 (30.6)	
**Dialysis Duration (hours)**	3.8 ± 0.2	3.8 ± 0.2	3.8 ± 0.3	0.307^a^
**Dialysis Vintage (months)**	36.7 ± 25.1	40.6 ± 25.9	32.4 ± 23.7	**0.02** ^**a**^
**Dialysis Frequency**				0.187^b^
Once a week	3 (1.7)	2 (2.1)	1 (1.2)	
Twice a week	145 (80.1)	72 (75.0)	73 (85.9)	
Thrice a week	33 (18.2)	22 (22.9)	11 (12.9)	
**Causes of ESRD**				0.199^b^
HTN	71 (39.2)	38 (39.6)	33 (38.8)	
DM	6 (3.3)	4 (4.2)	2 (2.4)	
HTN, DM	41 (22.7)	20 (20.8)	21 (24.7)	
CGN	10 (5.5)	9 (9.4)	1 (1.2)	
HTN, CGN	6 (3.3)	3 (3.1)	3 (3.5)	
Unknown	47 [26]	22 (22.9)	25 (29.4)	
**Household income**				0.563^b^
Poor	55 (30.4)	31 (32.3)	24 (28.2)	
Moderate	63 (34.8)	30 (31.3)	33 (38.8)	
High	63 (34.8)	35 (36.5)	28 (32.9)	
**Types of Dialyzers Used**	n = 152	n = 85	n = 67	**0.01** ^**b**^
Polysulfone	88 (57.9)	42 (49.4)	46 (68.7)	
Polynephrone	64 (42.1)	43 (50.6)	21 (31.3)	
**Types of Buffers Used**	(152)	n = 85	n = 67	0.576^b^
Bicarbonate	110 (72.4)	59 (69.4)	51 (76.1)	
Acetate	41 (27.0)	25 (29.4)	16 (23.9)	
Bicarbonate/Acetate	1 (0.7)	1 (1.2)	0	
**Kidney transplant**	n = 152	n = 85	n = 67	0.441^b^
Yes	1 (0.7)	0	1 (0.7)	
No	151 (99.3)	85 (100)	66 (98.5)	
**Diabetes Mellitus**	n = 156	n = 86	n = 70	0.984^b^
Yes	53 (34)	29 (33.7)	24 (34.3)	
No	103(66)	57 (66.3)	46 (65.7)	
**Tobacco use**				**0.004** ^**b**^
Yes	34 (18.8)	26 (27.1)	8 (9.4)	
No	147 (81.2)	70 (72.9)	77 (90.6)	
**Hepatitis C**	n = 152	n = 85	n = 67	0.801^b^
Yes	17 (11.2)	9 (10.6)	8 (11.9)	
No	135 (88.8)	76 (89.4)	59 (88.1)	

*Values are presented as mean ± SD for continuous variables and n (%) for categorical variables. HTN: Hypertension; DM: Diabetes Mellitus; CGN: Chronic glomerulonephritis. Continuous variables were analyzed using one-way ANOVA (superscript a), and categorical variables using Pearson’s χ*^2^ *test (superscript b).*

**Table 2:** Participant characteristics by reporting-plausibility group (n = 178).

Parameters	Acceptable reporters 116 (65.2)	Under reporters 57 (32.0)	Over reporters 5 (2.8)	*p*-Value
**Gender**				0.630^a^
Male	63 (67.7)	27 (29.0)	3 (3.2)	
Female	53 (62.4)	30 (35.3)	2 (2.4)	
**Age category**				0.647^a^
Young	25 (73.5)	8 (23.5)	1 (2.9)	
Middle	51 (60.7)	31 (36.9)	2 (2.4)	
Old	40 (66.7)	18 (30.0)	2 (3.3)	
**BMI category**				0.111^a^
BMI > 23	42 (58.3)	29 (40.3)	1 (1.4)	
BMI < 23	74 (69.8)	28 (26.4)	4 (3.8)	

*BMI: Body Mass Index. Data expressed as n (%) for categorical variables. Fisher’s exact test for categorical variables (superscript a)- which applied to every comparison involving the five over reporters. Reporting validity was determined using EI:BMR ratios: < 0.8 (under reporting), 0.8–1.3 (acceptable), and >1.3 (over reporting) applied to the Goldberg ratio adjusted for physical activity level.*

Hypertension was the predominant cause of ESRD (39.2%), followed by combined hypertension and diabetes (22.7%) and unknown etiologies (26%), with no significant gender variation. Household income distribution showed 30.4% in the low-income group, 34.8% moderate-income, and 34.8% high-income. Among 152 patients with available information, polysulfone dialyzers were used more frequently (57.9%) than polynephrone, with a significant sex difference. Bicarbonate-based dialysate was most common (72.4%). Kidney transplantation was rare (0.7%). Most participants were non-diabetic (66%) and non-reactive for hepatitis C (88.8%). Tobacco use differed significantly by sex, with higher prevalence among males.

Table 2 shows reporting validity based on EI:BMR, expressed as a proportion of the 178 participants with complete data. Acceptable reporting was observed in 65.2% of participants, while 32.0% underreported and 2.8% over reported. Underreporting was slightly more common among females. Middle-aged adults showed the highest proportion of underreporting. Most acceptable reporters had BMI < 23 kg/m^2^ (67.6%), whereas 43.8% of under reporters had BMI > 23 kg/m^2^. No significant associations were found between reporting category and sex, age group, or BMI. Because only five participants were classified as over reporters, all comparisons involving this group are statistically unstable and are interpreted descriptively throughout.

### Anthropometric and clinical parameters

Anthropometric characteristics are presented in Table 3. After excluding three patients with unmeasurable weight, the mean BMI of the cohort was 22.8 ± 4.3 kg/m^2^. Most patients (64.0%) had a normal BMI (18.5–24.9 kg/m^2^), while 13.5% were underweight, 16.9% overweight, and 5.6% obese. BMI differed significantly by sex, with women showing higher BMI values than men (*p* < 0.01). Sex differences were also observed for height, TSF, and MAC, with women demonstrating significantly greater TSF and MAC. No significant differences in BMI categories were observed across acceptable reporters, under-reporters, and over-reporters. However, TSF was significantly higher among under-reporters compared to the other groups (*p* = 0.03).

**Table 3:** Anthropometric characteristics of hemodialysis patients by sex and reporting accuracy.

Anthropometric Parameters	Total n = 178	Male	Female	p value	AR	UR	OR	*p-*Value
**Height (m)**	1.59 ± 0.22	1.65 ± 0.18	1.51 ± 0.24	**<0.001** ^**a**^	1.58 ± 0.27	1.60 ± 0.09	1.59 ± 0.10	0.861^a^
**Body weight (kg)**	59.2 ± 10.7	61.5 ± 8.8	56.6 ± 12.0	**<0.001** ^**a**^	58.7 ± 10.6	60.3 ± 10.9	55.4 ± 10.9	0.490^a^
**BMI (kg/m**^**2**^)	22.8 ± 4.3	22.0 ± 3.0	23.7 ± 5.2	**0.01** ^**a**^	22.4 ± 4.0	23.7 ± 4.6	22.0 ± 4.8	0.160^a^
**BMI category**				**<0.001** ^**b**^				0.851^c^
Under weight	24 (13.5)	13 (54.2)	11 (45.8)		17 (70.8)	5 (20.8)	1 (4.2)	
Normal weight	114 (64.0)	67 (58.8)	47 (41.2)		71 (62.3)	38 (33.3)	3 (2.6)	
Over weight	30 (16.9)	15 (50.0)	15 (50.0)		19 (63.3)	10 (33.3)	1 (3.3)	
Obese	10 (5.6)	0 (0.0)	10 (100.0)		6 (60.0)	4 (40.0)	0 (0.0)	
**TSF (mm)**	14.9 ± 7.8	11.6 ± 6.1	18.5 ± 7.8	**<0.001** ^**a**^	13.9 ± 7.1	17.2 ± 8.9	13.0 ± 5.3	**0.03** ^**a**^
**MAC (cm)**	26.4 ± 4.2	25.4 ± 3.2	27.4 ± 4.9	**<0.001** ^**a**^	26.0 ± 4.1	27.1 ± 4.4	25.8 ± 2.7	0.316^a^
**MAMC (cm)**	21.7 ± 2.5	21.8 ± 2.2	21.6 ± 2.9	0.636^a^	21.7 ± 2.6	21.7 ± 2.5	21.7 ± 2.6	0.998^a^

*AR: Acceptable reports; UR: Under reports; OR: Over reports; BMI: Body Mass Index; TSF: Triceps Skinfold; MAC: Mid-Arm Circumference; MAMC: Mid-Arm Muscle Circumference; Continuous variables are expressed as mean ± SD; categorical variables as n (%). Group differences were tested using ANOVA for continuous variables (superscript “a”) and Pearson’s χ*^2^ *test for categorical variables (superscript “b”), with Fisher’s exact test substituted where any expected cell count was below five (superscript “c”).*

Clinical and biochemical parameters are summarized in **Table 4**. Sodium, glucose, and serum TIBC tended to be higher among over-reporters, but no clinical parameter differed significantly across reporting-accuracy groups. Missing biochemical data were excluded when not available in medical records.

**Table 4:** Clinical and biochemical parameters of hemodialysis patients according to reporting accuracy.

Clinical Parameters	Total	AR	UR	OR	*p-*Value
Sodium (mmol/L) n = 177	135.6 ± 4.6	135.4 ± 5.0	136.1 ± 3.6	136.1 ± 4.9	0.625^a^
Potassium (mmol/L) n = 176	4.72 ± 0.97	4.77 ± 1.01	4.63 ± 0.90	4.45 ± 0.83	0.545^a^
Albumin (g/dl) n = 176	3.62 ± 0.43	3.58 ± 0.43	3.69 ± 0.43	3.72 ± 0.29	0.224^a^
Serum TIBC (mcg/dl) n = 176	235.4 ± 61.9	234.2 ± 64.5	237.2 ± 59.3	241.3 ± 30.4	0.936^a^
Creatinine (µmol/L) n = 178	912.6 ± 386.5	946.0 ± 425.3	850.5 ± 303.8	845.2 ± 149.4	0.290^a^
Hemoglobin (g/dl) n = 178	8.93 ± 1.80	8.90 ± 1.85	9.01 ± 1.67	8.66 ± 2.30	0.885^a^
MDRD Calculated eGFR (ml/min/1.73m^2^) n = 178	5.76 ± 2.59	5.69 ± 2.57	5.92 ± 2.73	5.56 ± 0.82	0.840^a^
Glucose (mg/dl) n = 178	142.6 ± 50.0	141.7 ± 49.5	146.9 ± 52.4	114.1 ± 21.3	0.352^a^

*MDRD eGFR: Estimated Glomerular Filtration Rate calculated using the MDRD equation; Continuous variables are presented as mean ± SD. ANOVA was used for continuous variables (superscript “a”).*

### Macronutrient intake

Macronutrient intake patterns are summarized in Table 5. Under-reporters had the lowest mean energy intake (1164 ± 209 kcal/day), whereas over-reporters had the highest (1798 ± 478 kcal/day). Similar trends were observed for protein, carbohydrate, and fat intake, with significant differences among reporting groups (*p* < 0.001).

**Table 5:** Macronutrient intake of hemodialysis patients by reporting accuracy.

Macronutrient	Total n = 178	AR	UR	OR	p-Value	NKF KDOQI Guideline
Energy (kcal)	1475.0 ± 322.7	1614.0 ± 242.8	1163.8 ± 209.2	1798.3 ± 477.8	**<0.001** ^**a**^	Based on body weight
EI: BMR (adjusted for PAL)	0.89 ± 0.23	0.98 ± 0.12	0.66 ± 0.11	1.64 ± 0.38	**<0.001** ^**a**^	
Carbohydrate (g)	205.2 ± 48.7	223.3 ± 40.4	165.3 ± 36.0	240.4 ± 78.2	**<0.001** ^**a**^	Based on body weight
Protein (g)	56.7 ± 14.1	61.7 ± 12.1	45.6 ± 11.1	66.3 ± 17.5	**<0.001** ^**a**^	Based on body weight
Fat (g)	44.0 ± 14.0	48.7 ± 12.0	32.9 ± 9.2	60.1 ± 24.1	**<0.001** ^**a**^	Based on body weight
DEI (kcal/kg/body wt./day)	23.6 ± 5.4	26.1 ± 3.8	17.9 ± 3.0	34.3 ± 3.7	**<0.001** ^**a**^	30-35 Kcal/Kg body wt./day
DPI (g/kg body wt./day)	0.90 ± 0.24	1.00 ± 0.21	0.70 ± 0.16	1.09 ± 0.13	**<0.001** ^**a**^	1.0-1.2 g/ Kg body wt./day
Phosphorous: Protein (mg/g)	14.7 ± 1.7	14.7 ± 1.6	14.9 ± 1.9	14.2 ± 2.6	0.518^a^	<12 mg/g
Dietary Cholesterol (mg/day)	56.7 (7.5-113.4)	60.3 (14.0-112.8)	53.2 (0.2-124.1)	29.2 (12.3-53.2)	0.307^b^	<200 mg
SFA(g/day)	1.53 ± 1.30	1.68 ± 1.31	1.13 ± 0.99	2.67 ± 2.59	**<0.001** ^**a**^	**–**
MUFA(g/day)	1.38 ± 1.07	1.48 ± 0.99	1.13 ± 1.18	1.97 ± 1.11	0.059^a^	**–**
PUFA(g/day)	0.54 (0.35-0.95)	0.55 (0.40-0.96)	0.50 (0.22-0.80)	1.28 (0.59-1.38)	**0.019** ^**b**^	**–**

*EI: Energy Intake; BMR: Basal Metabolic Rate; DEI: Dietary Energy Intake; DPI: Dietary Protein Intake; NKF KDOQI: National Kidney Foundation Kidney Disease Outcomes Quality Initiative; SFA: Saturated Fatty Acids; MUFA: Monounsaturated Fatty Acids; PUFA: Polyunsaturated Fatty Acids; Continuous variables are expressed as mean ± SD or median (IQR). Differences across reporting groups (AR: acceptable, UR: underreport, OR: over report) were analyzed using ANOVA (superscript “a”) and Kruskal-Wallis test (superscript “b”).*

The average dietary protein intake (DPI) and dietary energy intake (DEI) were 0.90 ± 0.24 g/kg/day and 23.6 ± 5.4 kcal/kg/day, respectively, both below KDOQI recommendations (1.0–1.2 g/kg/day and 30–35 kcal/kg/day). Intakes of saturated, monounsaturated and polyunsaturated fatty acids were low, with a significant difference across reporting groups for saturated and polyunsaturated fatty acids.

### Micronutrient intake

Vitamin and mineral intakes are presented in full in S1 and S2 Tables in S1 File, and the proportion of patients meeting each target is shown in Fig 1.

**Fig 1:**
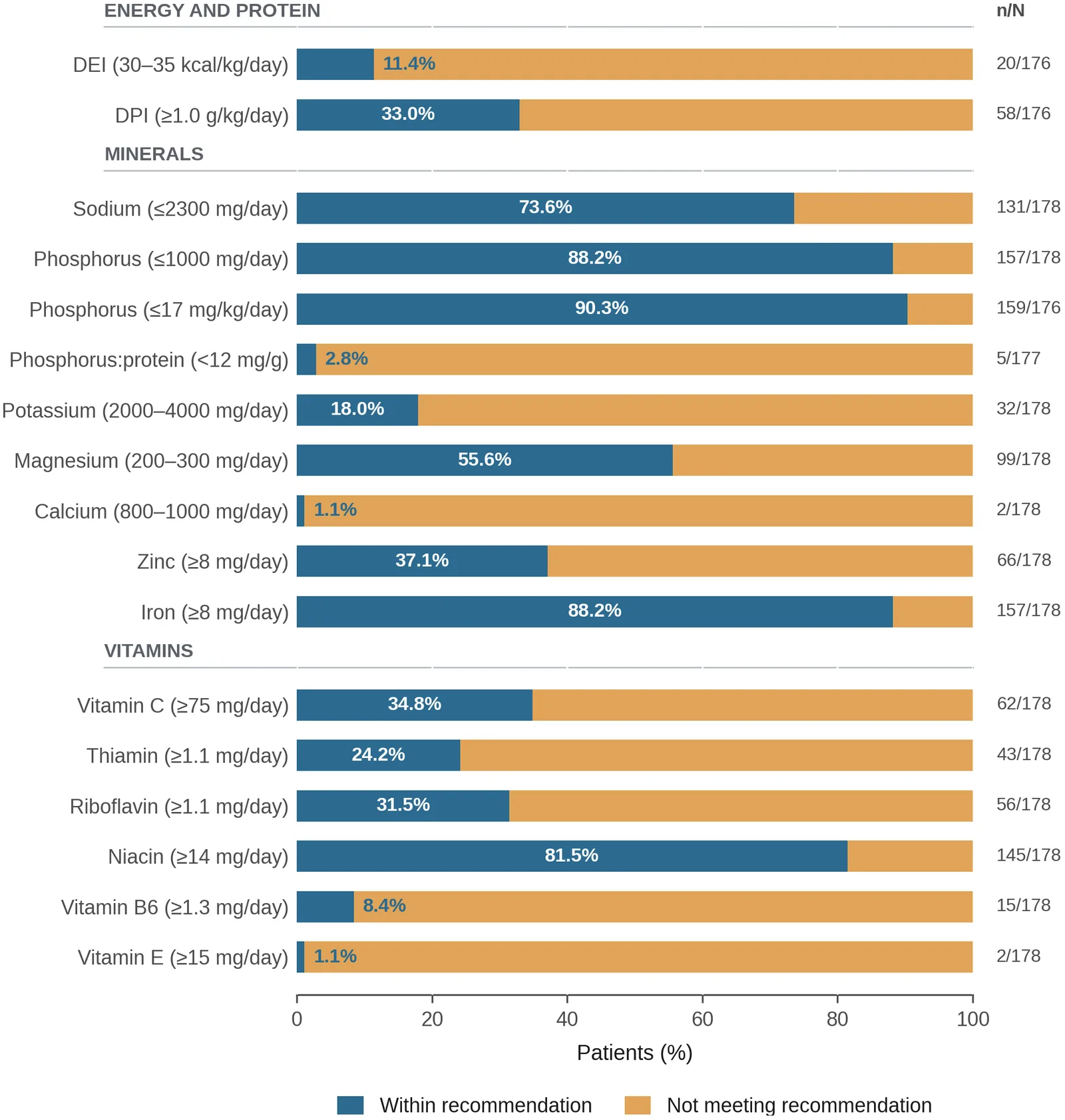
Compliance of hemodialysis patients with KDOQI dietary recommendation.

Mean intakes of every vitamin examined except niacin fell below the corresponding dietary reference intake. Niacin was the only vitamin reached by most participants (145 of 178, 81.5%). Vitamin C, thiamin and riboflavin were reached by a minority (34.8%, 24.2% and 31.5% respectively), and vitamins A, D, E, K, B6, B12 and folate by almost no one. Reported intakes differed significantly across reporting-plausibility groups for vitamins A, C, E and K, thiamin, riboflavin, niacin, vitamin B6 and folate, but not for vitamin D, vitamin B12 or pantothenic acid, with under-reporters consistently lowest. The apparent near-total shortfall in vitamins A, D, K and B12 should be interpreted with caution: median intakes of these nutrients are implausibly low for a diet containing leafy vegetables, small fish and milk, which points to incomplete micronutrient coverage in the food composition data for some local foods rather than to true intake near zero. This is considered further in the Discussion.

Intakes of phosphorus, sodium and iron were within recommended limits for most participants, whereas calcium (2 of 178, 1.1%), potassium (32 of 178, 18.0%) and zinc (66 of 178, 37.1%) were not. The phosphorus-to-protein ratio exceeded the recommended 12 mg/g in all but five participants. Every mineral except iron differed significantly across reporting-plausibility groups, under-reporters consistently recording the lowest intakes.

#### Adherence to dietary targets and the effect of plausibility screening.

Weight-adjusted intake data were available for 176 participants. In the cohort as a whole, 20 (11.4%) achieved the recommended energy intake of 30–35 kcal/kg/day, 58 (33.0%) reached the lower bound of the protein recommendation (≥1.0 g/kg/day) and 16 (9.1%) reached its upper bound (≥1.2 g/kg/day). Eighteen participants (10.2%) met the energy target and the lower-bound protein target simultaneously, and nine (5.1%) met the energy target and the upper-bound protein target simultaneously.

Restricting the analysis to participants whose reported intake was plausible changed these estimates substantially, and in opposite directions according to the type of target (Table 6 and Fig 2). For targets expressed as a minimum intake, adherence was higher among plausible reporters than in the pooled cohort: energy 15.5% versus 11.4%, protein at the 1.0 g/kg/day threshold 47.4% versus 33.0%, and zinc 50.0% versus 37.1%. For targets expressed as an upper limit the reverse held: apparent adherence to the sodium limit was 65.5% among plausible reporters versus 73.6% in the pooled cohort, and to the 1000 mg/day phosphorus limit 84.5% versus 88.2%. Every contrast between plausible and invalid reporters was statistically significant (Table 6).

**Table 6:** Estimated adherence to dietary targets in the whole cohort, among plausible reporters and among invalid reporters.

Target	Whole cohort, n/N (%)	Plausible reporters, n/N (%)	Invalid reporters, n/N (%)	Difference, plausible − whole cohort (pp)	p
Energy, DEI 30–35 kcal/kg/day	20/176 (11.4)	18/116 (15.5)	2/60 (3.3)	+4.2	0.022
Protein, DPI ≥ 1.0 g/kg/day	58/176 (33.0)	55/116 (47.4)	3/60 (5.0)	+14.5	<0.001
Protein, DPI ≥ 1.2 g/kg/day	16/176 (9.1)	15/116 (12.9)	1/60 (1.7)	+3.8	0.012
Sodium ≤2300 mg/day	131/178 (73.6)	76/116 (65.5)	55/62 (88.7)	−8.1	<0.001
Phosphorus ≤1000 mg/day	157/178 (88.2)	98/116 (84.5)	59/62 (95.2)	−3.7	0.049
Phosphorus ≤17 mg/kg/day	159/176 (90.3)	100/116 (86.2)	59/60 (98.3)	−4.1	0.013
Potassium 2000–4000 mg/day	32/178 (18.0)	29/116 (25.0)	3/62 (4.8)	+7.0	<0.001
Zinc ≥8 mg/day	66/178 (37.1)	58/116 (50.0)	8/62 (12.9)	+12.9	<0.001

*Invalid reporters comprise under- and over-reporters combined. p values are from Fisher’s exact test comparing plausible with invalid reporters. Positive differences indicate that restricting analysis to plausible reporters raises estimated adherence. Note the change of sign between intake targets (energy, protein, potassium, zinc) and restriction targets (sodium, phosphorus). pp, percentage points.*

**Fig 2:**
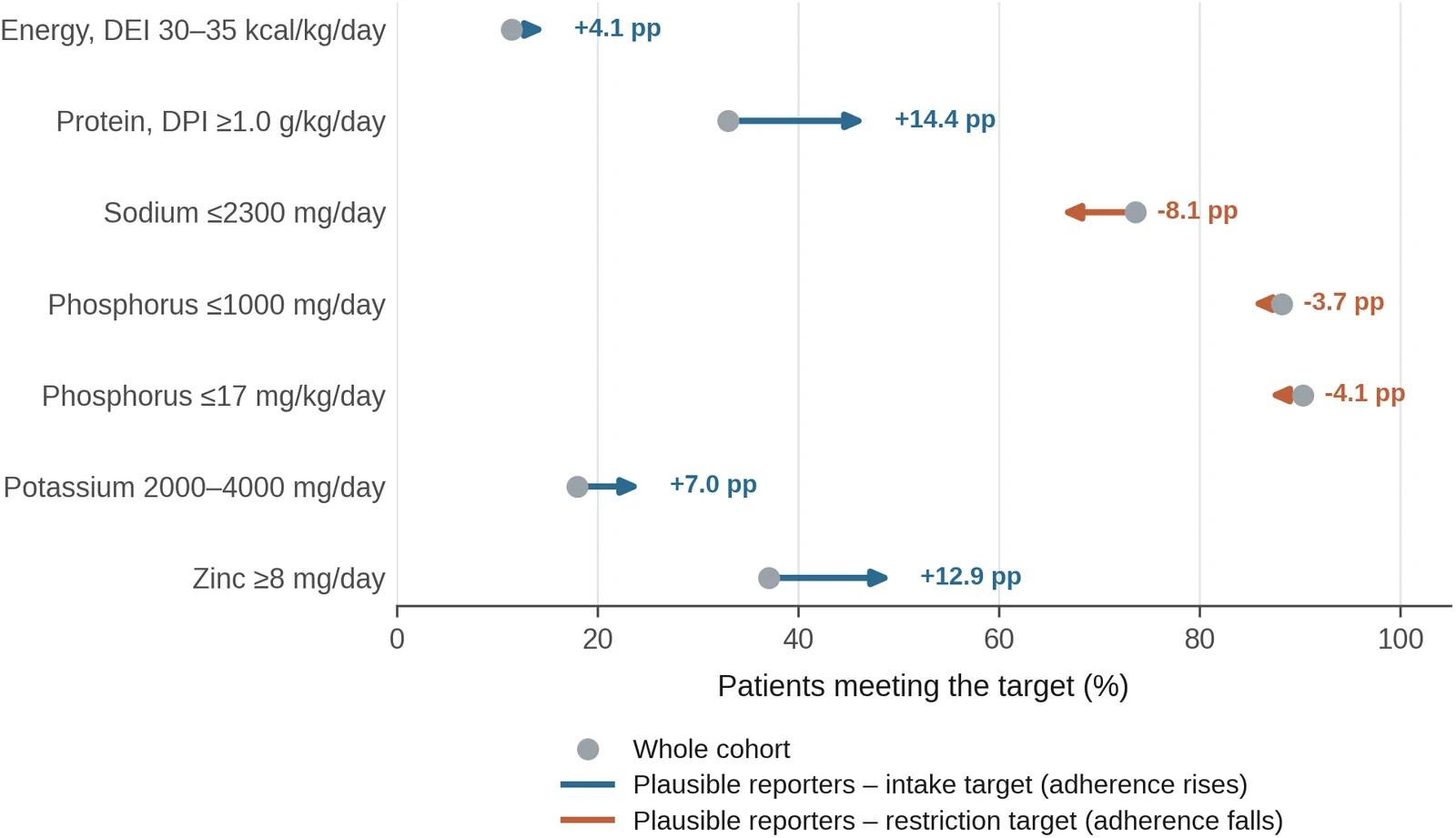
Effect of plausibility screening on estimated adherence. Screening raises estimated adherence to intake targets and lowers it for restriction targets.

#### Plausible versus invalid reporters (sensitivity analysis).

Because only five participants were classified as over reporters, a pre-specified sensitivity analysis compared valid reporters (n = 116) with all invalid reporters (under- and over reporters combined, n = 62). Invalid reporters recorded substantially lower intakes of energy (1614.0 ± 242.8 vs 1214.9 ± 292.4 kcal/day, p < 0.001), protein (61.7 ± 12.1 vs 47.3 ± 12.8 g/day, p < 0.001) and weight-adjusted energy (26.1 ± 3.8 vs 18.7 ± 4.7 kcal/kg/day, p < 0.001). The same direction of difference was seen for calcium, phosphorus, zinc, sodium and potassium (all p < 0.001). Among anthropometric measures, triceps skinfold thickness was significantly greater in invalid reporters (16.9 ± 8.7 vs 13.9 ± 7.1 mm, p 0.03), whereas BMI and serum albumin did not differ significantly. This analysis supports the conclusions drawn about misreporting without relying on the unstable five-participant overreporting subgroup.

#### Predictors of under-reporting.

In multivariable logistic regression (S3 Table in S1 File), longer dialysis vintage was the only independent predictor of under-reporting (adjusted odds ratio 0.81 per additional 12 months, 95% CI 0.67–0.96, p = 0.019), indicating that patients newer to dialysis were more likely to under-report. Female sex (1.12, 95% CI 0.55–2.28, p = 0.753), age (1.08 per decade, p = 0.541), BMI (1.05 per kg/m^2^, p = 0.230), poor appetite (1.29, p = 0.473) and twice-weekly dialysis (0.66, p = 0.378) were not independently associated with under-reporting, and the model explained little of the variance (McFadden pseudo-R^2^ = 0.053). The statement in the original submission that under-reporting was more common among women and among patients with higher BMI is therefore not supported on adjustment and has been corrected.

#### Dialysis frequency.

One hundred and forty-five participants (80.1%) received twice-weekly dialysis and 33 (18.2%) thrice-weekly. Reported energy intake (1485.8 ± 339.4 versus 1424.1 ± 247.4 kcal/day, p = 0.236), protein intake (p = 0.696), weight-adjusted energy (p = 0.722) and weight-adjusted protein (p = 0.788) did not differ by dialysis frequency, nor did the prevalence of poor appetite (p = 0.547) or of invalid reporting (p = 0.998). Patients dialysing three times weekly had higher serum albumin (37.6 ± 3.6 versus 35.9 ± 4.4 g/L, p = 0.021) and higher BMI (24.6 ± 5.5 versus 22.4 ± 3.9 kg/m^2^, p = 0.042). Full results are given in S4 Table in S1 File.

#### Appetite and dietary intake.

Appetite assessment using the Appetite and Diet Assessment Tool (ADAT) revealed critical insights into the dietary behavior of Bangladeshi hemodialysis patients (Table 7). The majority of patients (42.0%) exhibited poor appetite, 34.8% had fair appetite, and only 23.2% reported good appetite. Females were disproportionately affected, with more than half reporting poor appetite, highlighting a gender-related vulnerability in dietary adherence.

**Table 7:** Appetite, Anthropometry, and Dietary Assessment Based on ADAT Scores (n = 181).

Parameters	Good 42 (23.2%)	Fair 63 (34.8%)	Poor 76 (42.0%)	*p-Value*
**Gender**				**0.02** ^**b**^
*Male*	23 (24.0)	41 (42.7)	32 (33.3)	
*Female*	19 (22.4)	22 (25.9)	44 (51.8)	
**Anthropometry**				
*Weight (kg)*	58.3 ± 11.0	60.1 ± 10.4	58.8 ± 10.7	0.674^a^
*BMI kg/m* ^*2*^	21.9 ± 3.9	23.0 ± 4.0	23.1 ± 4.6	0.322^a^
**BMI category**				0.706^c^
*Underweight*	7 (29.2)	7 (29.2)	10 (41.7)	
*Normal weight*	28 (24.6)	38 (33.3)	48 (42.1)	
*Overweight*	6 (20.0)	14 (46.7)	10 (33.3)	
*Obese*	1 (10.0)	3 (30.0)	6 (60.0)	
**Dietary assessments**				
*Energy (kcal)*	1375.2 ± 334.2	1550.9 ± 302.5	1469.1 ± 320.2	**0.02** ^**a**^
*Protein (g)*	55.2 ± 17.7	58.1 ± 12.3	56.3 ± 13.3	0.570^a^
*DEI (kcal/kg/day)*	22.2 ± 6.1	24.6 ± 4.5	23.4 ± 5.5	0.081^a^
*DPI (g/kg/day)*	0.89 ± 0.3	0.92 ± 0.19	0.90 ± 0.24	0.753^a^
*EI: BMR*	0.82 ± 0.22	0.93 ± 0.17	0.91 ± 0.27	0.060^a^

*ADAT: Appetite and Diet Assessment Tool; BMI: Body Mass Index; EI: Energy Intake; BMR: Basal Metabolic Rate; DEI: Dietary Energy Intake; DPI: Dietary Protein Intake; NS: Not Significant. Data are expressed as mean ± SD for continuous variables and n (%) for categorical variables. Differences in continuous variables were analysed using ANOVA (superscript a), while categorical variables were evaluated using Pearson χ*^2^ *test (superscript b). Fisher’s exact test (superscript “c”). Appetite was graded from the ADAT summary score (good ≤2, fair = 3, poor ≥4)*

Contrary to expectation, self-rated appetite did not correspond to lower measured intake. Reported energy intake differed significantly across appetite categories (F (2, 175) = 3.83, p = 0.024) but not monotonically: the fair-appetite group recorded the highest mean intake (1550.9 ± 302.5 kcal/day), followed by the poor-appetite group (1469.1 ± 320.2 kcal/day), with the good-appetite group lowest (1375.2 ± 334.2 kcal/day). In Holm-adjusted pairwise comparisons only the good versus fair contrast was significant (p = 0.024); good versus poor and fair versus poor were not (both p = 0.258). Protein intake, weight-adjusted energy and protein, BMI and the EI:BMR ratio did not differ across appetite categories. Appetite rating was, however, associated with reporting plausibility: invalid reporters were more likely than plausible reporters to rate their appetite at either extreme, while fair ratings were concentrated among plausible reporters (χ^2^(2) = 7.50, p = 0.024).

Fig 3 examines patients’ appetite ratings in relation to reporting accuracy. Patients with lower appetites tended to underreport dietary intake, with 44.3% of under reporters exhibiting poor appetite, whereas over-reporters predominantly reported adequate intake despite high energy consumption. No significant differences in appetite were observed between valid and invalid reporters overall, although most valid reporters indicated a fair appetite.

**Fig 3:**
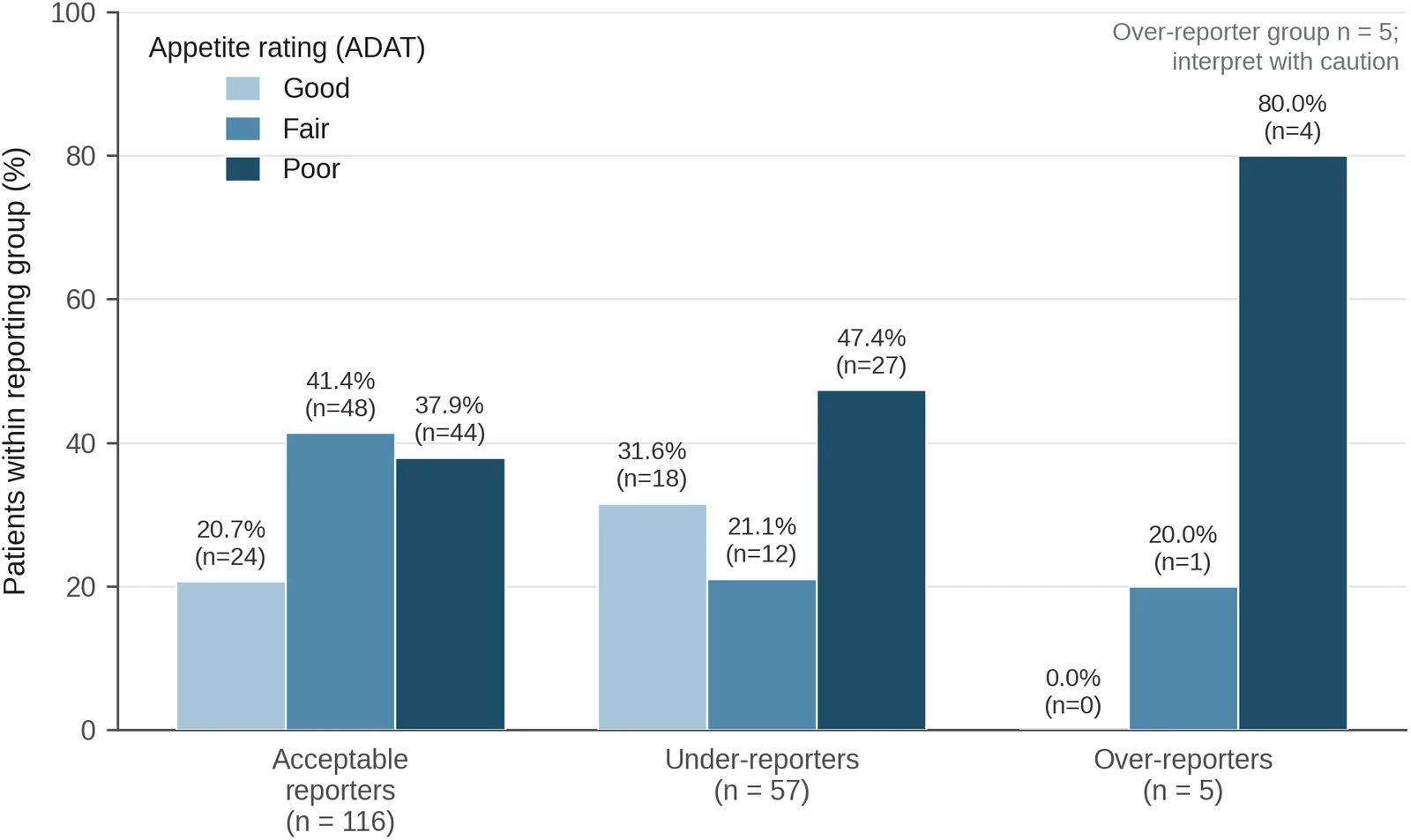
Appetite rating by reporting plausibility. The over-reporter group contains five participants and is shown for completeness only.

## Discussion

This study represents the first comprehensive evaluation of dietary intake and nutritional compliance among hemodialysis patients in Bangladesh using validated software incorporating local and ethnic recipes, incorporating macronutrient, micronutrient, mineral, appetite, anthropometric, and clinical data, while correcting for misreporting via the EI:BMR ratio. Three findings stand out. Patients fell well short of KDOQI energy and protein targets an expected result. Less expected, a third reported intake incapable of sustaining their own body weight, and appetite ratings failed to flag the patients eating least. Together, these findings raise a question that is as much about the instrument as the population: in a setting where dietary recall is the only available assessment, how much of the measured deficit reflects diet, and how much reflects measurement?

Under-reporting was common (32.0%) and over-reporting rare (2.8%), consistent with the wider dietary assessment literature and with prior work in maintenance hemodialysis [25,26]. The effect on this cohort was not subtle: under-reporters recorded a weight-adjusted energy intake of just 17.9 ± 3.0 kcal/kg/day, barely half the KDOQI lower bound. Sustained, that level of intake is incompatible with weight stability and since these patients were clinically stable with a BMI no different from plausible reporters, the simpler explanation is a wrong record, not a starving patient.

Renal dietary guidance sets two kinds of target: energy and protein are floors to reach, sodium, phosphorus and potassium are ceilings not to cross. A patient who under-records food fails the first and appears to ace the second, the same error, cutting both ways. Restricting our analysis to plausible reporters raised protein adherence from 33.0% to 47.4% and energy adherence from 11.4% to 15.5%, while lowering apparent sodium adherence from 73.6% to 65.5% and phosphorus from 88.2% to 84.5%. A clinician reading an unscreened recall would push protein on a patient who may not need it, while feeling reassured about a sodium intake that may in fact be high. This bidirectional bias has not, to our knowledge, been quantified before in hemodialysis and it costs nothing to screen for, since the check needs only the height, weight and age already on file.

Under-reporters also had greater triceps skinfold thickness than plausible reporters, echoing the familiar link between adiposity and misreporting [15,27]. But BMI itself didn’t differ, and on adjustment neither BMI nor sex predicted under-reporting only shorter dialysis vintage did. Our guess is exposure: patients followed for years have been asked about their diet often enough to answer it well; new patients haven’t. The model explained little variance overall, which if anything argues for screening every recall rather than targeting suspicion at particular patients. And the split between skinfold thickness and BMI is worth keeping rather than smoothing over: fluid status confounds weight in dialysis, so a normal BMI can hide both fat excess and muscle loss. This cohort’s largely normal BMI should not be mistaken for adequate nutrition.

The appetite findings were the clearest departure from expectation. Poor appetite was common (42.0%) and fell hardest on women [17,31], but it tracked nothing. Energy intake was lowest in the good-appetite group; the one significant difference across categories was driven by fair-appetite patients eating the most. A single-item rating asks how someone feels about eating, not how much they ate, and in uraemia those can genuinely diverge shaped by taste change, gut symptoms, inflammation and post-dialysis fatigue rather than food actually consumed [32–35]. We can’t rule out something less comfortable either: invalid reporters were more likely to rate their appetite at either extreme (p = 0.024), suggesting appetite rating and recall accuracy may share a common reporting bias. Either way, appetite should not stand in for measured intake here, and a good rating is no reassurance. That doesn’t undercut its prognostic value elsewhere [36]; it separates that value from any claim about consumption.

Micronutrient intakes were below reference values for almost every nutrient examined, with niacin the sole exception, and we would urge caution in how far this is taken. Prescribed renal vitamin supplements were not captured, which compounds the underestimate. Stating this openly seems preferable to reporting a deficiency prevalence we could not defend. One mineral finding is robust and clinically actionable. The phosphorus-to-protein ratio exceeded 12 mg/g in all but five participants, while absolute phosphorus intake was within the recommended limit for 88.2%. Read together these say that patients are obtaining phosphorus without the protein that should accompany it, the least favourable combination in dialysis nutrition. The counselling implication is specific and inverts the usual message: the target is phosphorus-dense, protein-poor foods, particularly processed items containing inorganic phosphate additives, rather than restriction of protein-rich foods, which these patients are already eating too little of.

Four-fifths of the cohort dialysed twice weekly, common across South Asia and driven by cost rather than clinical choice [37,38]; reduced frequency has been linked to higher mortality [39]. Hypertension, alone or with diabetes, was the leading cause of ESRD, matching earlier Bangladeshi reports [16]. Since KDOQI targets were derived mostly from thrice-weekly populations [40–42], we expected the twice-weekly schedule via persistent uraemia and gut symptoms to explain part of the low adherence. It didn’t: energy, protein, weight-adjusted intake, appetite and reporting plausibility were all similar across frequencies. That contrasts with Nieves-Anaya and colleagues, who found worse nutrition and hydration among twice-weekly patients in a similar setting [43]; the difference may reflect our small thrice-weekly group (n = 33) or our lack of residual urea clearance data. Taken at face value, the shortfall here looks less like a consequence of the dialysis prescription and more like one of food access, cost, and the near-absence of renal dietetic support.

Clinically, the picture is less reassuring than it looks. Mean albumin was 36.2 ± 4.3 g/L, consistent with moderate compromise [44]; haemoglobin averaged 89 ± 18 g/L; potassium sat safely within range at 4.72 ± 0.97 mmol/L [45]. Yet mean BMI was 22.8 kg/m^2^ and most patients were “normal weight.” A cohort can look unremarkable on the number clinicians check first while eating at two-thirds of requirement and that combination of normal weight, low albumin, low intake and an uninformative appetite rating is exactly how protein-energy wasting goes unnoticed [13,46], as seen in other hemodialysis cohorts [47–49].

Collectively, these findings support a modest and specific set of recommendations for dialysis units in resource-limited settings. Dietary recall should be screened for physiological plausibility before it informs any counselling decision. Apparent compliance with sodium and phosphorus restriction should be treated with particular scepticism in patients whose reported energy intake is implausibly low. Self-rated appetite should be recorded, because it carries prognostic information of its own, but interpreted alongside rather than in place of measured intake. And phosphorus counselling should target the source of phosphorus rather than the quantity of protein. None of these requires equipment, funding or staff that these units do not already have.

### Strengths and limitations

This study provides the first comprehensive evaluation of macro- and micronutrient intake among Bangladeshi hemodialysis patients using validated, locally adapted dietary analysis software. Classifying participants as valid, under-, or over-reporters strengthened the accuracy of dietary assessment, while the combined analysis of anthropometry, appetite, and clinical markers enabled a well-rounded understanding of nutritional status.

The study’s small sample size limits its generalizability, and reliance on 24-hour recalls may have introduced misreporting. Variation in household utensils and recipe preparation could contribute to measurement error, and fluctuations in patients’ health may have affected data quality. Limited access to trained renal dietitians also posed logistical constraints. Several further limitations should be acknowledged. Participants were enrolled by consecutive convenience sampling from four hospital-based units, so patients dialysing in smaller private centres or unable to afford regular dialysis are unlikely to be represented, and the sample should not be treated as nationally representative. Only five participants met the criterion for overreporting, and comparisons involving this group are correspondingly unstable. No correction for multiple comparisons was applied across the large number of nutrients examined, so isolated significant results require replication. The Goldberg cut-offs were derived in weight-stable general populations and have not been formally validated in maintenance hemodialysis, where fluid shifts, altered resting energy expenditure and the use of predictive BMR equations may all bias classification. All analyses reported here have been recomputed from the primary dataset, and a small number of values differ from those in the original submission; the recomputed figures should be regarded as authoritative. Finally, the cross-sectional design precludes causal inference about the relationships between appetite, dietary intake and nutritional status.

## Conclusion

Nutrition is central to the care of people receiving maintenance hemodialysis. Although dialysis sustains life, it does not erase the profound metabolic and dietary vulnerabilities created by chronic kidney disease. Our findings reveal a striking mismatch between actual dietary intake and KDOQI recommendations: many patients consumed substantially lower or higher amounts of energy and protein than required, underscoring a silent but critical nutritional burden that often goes unnoticed in routine clinical care.

By rigorously differentiating valid, under-, and over-reporters, and by integrating anthropometric, appetite, and dietary markers, this study offers one of the most comprehensive nutritional profiles of Bangladeshi hemodialysis patients to date. Importantly, we show that reliance on unfiltered self-reported dietary data can mask true deficiencies and misinform clinical decisions, particularly for energy, protein, fat, calcium, and zinc intake. The observed dissociation between subjective appetite ratings and measured dietary intake further highlights the need for systematic appetite assessment, interpreted alongside objective intake data, as part of regular dialysis care.

Together, these findings reveal an urgent gap in renal nutrition services and call for structured dietetic counseling tailored to the unique needs of this population. Beyond documenting a national problem, this study sets the stage for future work including intervention trials, culturally adapted dietary tools, and policy-level strategies to ensure that every patient receiving dialysis not only survives, but thrives. In a setting where renal nutrition care is still emerging, this work provides a foundation upon which future researchers and clinicians can build a more equitable and informed approach to kidney health.

## Supporting information

S1 File Supplementary tables. (DOCX)

S1 Dataset. Minimal anonymised dataset underlying the findings reported in this manuscript.
(XLSX)
