# Supplementary material for: Dietary intake patterns and KDOQI guideline compliance among hemodialysis patients in Bangladesh: Gaps, misreporting, and clinical implications
Source: PLoS One. 2026 Oct 12;21(10):e0360654. doi: 10.1371/journal.pone.0360654 (PMC13657316; doi:10.1371/journal.pone.0360654)
Supplement: Supplementary file 1 [file pone.0360654.s001.docx]

**Dietary intake patterns and KDOQI guideline compliance among hemodialysis patients in Bangladesh: gaps, misreporting, and clinical implications**

**Supporting Information**

**S1 Table. Vitamin intake by reporting-plausibility group (n = 178).**

| **Nutrient** | **Total** | **Acceptable-rep.** | **Under-rep.** | **Over-rep.** | **p** | **Reference intake** |
| --- | --- | --- | --- | --- | --- | --- |
| Vitamin A (IU/day) | 539.8 ± 365.2 | 594.2 ± 377.7 | 415.5 ± 305.8 | 692.9 ± 392.9 | 0.006 | ≥2333 IU (700–900 µg RAE) |
| Vitamin D (IU/day) | 4.40 (0.65–8.80) | 4.40 (1.38–8.80) | 4.40 (0.00–8.80) | 1.63 (1.62–4.40) | 0.334 | ≥600 IU |
| Vitamin E (mg/day) | 7.39 ± 2.34 | 8.25 ± 1.88 | 5.46 ± 1.39 | 9.47 ± 5.93 | <0.001 | ≥15 mg |
| Vitamin K (µg/day) | 2.39 (1.61–3.84) | 2.60 (1.88–4.15) | 1.77 (0.95–2.53) | 7.76 (2.93–11.28) | <0.001 | ≥90 µg |
| Vitamin C (mg/day) | 72.3 ± 48.9 | 77.3 ± 45.7 | 58.5 ± 42.3 | 114.0 ± 123.3 | 0.008 | ≥75 mg |
| Thiamin (mg/day) | 0.92 ± 0.29 | 1.02 ± 0.28 | 0.72 ± 0.20 | 0.90 ± 0.28 | <0.001 | ≥1.1 mg |
| Riboflavin (mg/day) | 0.99 ± 0.37 | 1.03 ± 0.37 | 0.89 ± 0.32 | 1.35 ± 0.38 | 0.006 | ≥1.1 mg |
| Niacin (mg/day) | 18.7 ± 5.1 | 20.7 ± 4.1 | 14.2 ± 3.6 | 22.0 ± 9.1 | <0.001 | ≥14 mg |
| Vitamin B6 (mg/day) | 0.97 ± 0.98 | 1.14 ± 1.16 | 0.65 ± 0.22 | 0.88 ± 0.36 | 0.008 | ≥1.3 mg |
| Folate (µg/day) | 132.1 ± 51.6 | 143.2 ± 53.3 | 107.8 ± 38.6 | 151.1 ± 54.3 | <0.001 | ≥400 µg DFE |
| Vitamin B12 (µg/day) | 0.20 (0.06–0.41) | 0.21 (0.07–0.42) | 0.19 (0.04–0.39) | 0.15 (0.07–0.16) | 0.713 | ≥2.4 µg |
| Pantothenic acid (mg/day) | 0.44 ± 0.40 | 0.46 ± 0.44 | 0.39 ± 0.32 | 0.44 ± 0.49 | 0.561 | ≥5 mg |

*Values are mean ± SD or median (IQR); groups compared by one-way ANOVA. Reference values are age- and sex-appropriate dietary reference intakes, not the supplementation doses recommended for dialysis patients; folate and vitamin B6 are therefore benchmarked at 400 µg/day and 1.3 mg/day.*

**S2 Table. Mineral intake by reporting-plausibility group (n = 178).**

| **Mineral** | **Total** | **Acceptable-rep.** | **Under-rep.** | **Over-rep.** | **p** | **Reference intake** |
| --- | --- | --- | --- | --- | --- | --- |
| Calcium (mg/day) | 387.1 ± 174.8 | 420.2 ± 175.7 | 307.1 ± 112.5 | 530.5 ± 371.9 | <0.001 | 800–1000 mg |
| Phosphorus (mg/day) | 812.7 ± 179.6 | 883.8 ± 147.3 | 658.5 ± 138.7 | 918.9 ± 187.3 | <0.001 | ≤1000 mg |
| Phosphorus (mg/kg/day) | 13.1 ± 3.1 | 14.3 ± 2.6 | 10.4 ± 2.1 | 16.8 ± 0.5 | <0.001 | ≤17 mg/kg |
| Sodium (mg/day) | 1967.7 ± 659.9 | 2153.5 ± 596.2 | 1566.4 ± 601.7 | 2231.2 ± 772.9 | <0.001 | ≤2300 mg |
| Potassium (mg/day) | 1586.3 ± 429.2 | 1768.7 ± 337.8 | 1190.4 ± 289.4 | 1869.0 ± 633.3 | <0.001 | 2000–4000 mg |
| Magnesium (mg/day) | 250.8 ± 63.7 | 275.7 ± 55.6 | 198.1 ± 41.2 | 273.9 ± 92.4 | <0.001 | 200–300 mg |
| Iron (mg/day) | 11.68 (9.27–14.97) | 12.61 (10.57–15.40) | 8.95 (7.74–11.72) | 14.04 (10.54–21.63) | <0.001 | ≥8 mg |
| Zinc (mg/day) | 7.31 ± 2.16 | 8.13 ± 1.89 | 5.51 ± 1.44 | 8.68 ± 2.54 | <0.001 | ≥8 mg |

*Values are mean ± SD; groups compared by one-way ANOVA. Weight-adjusted values use post-dialysis (dry) body weight and are available for 176 participants.*

**S3 Table. Unadjusted and adjusted predictors of under-reporting.**

| **Predictor** | **Unadjusted OR (95% CI)** | **p** | **Adjusted OR (95% CI)** | **p** |
| --- | --- | --- | --- | --- |
| Female sex | 1.40 (0.74–2.67) | 0.302 | 1.12 (0.55–2.28) | 0.753 |
| Age (per 10 years) | 1.13 (0.89–1.44) | 0.310 | 1.08 (0.84–1.38) | 0.541 |
| BMI (per 1 kg/m²) | 1.07 (0.99–1.16) | 0.071 | 1.05 (0.97–1.14) | 0.230 |
| Poor appetite (ADAT) | 1.47 (0.77–2.80) | 0.249 | 1.29 (0.65–2.55) | 0.473 |
| Dialysis vintage (per 12 months) | 0.81 (0.68–0.96) | 0.014 | 0.81 (0.67–0.96) | 0.019 |
| Twice-weekly dialysis | 0.78 (0.34–1.78) | 0.547 | 0.66 (0.26–1.66) | 0.378 |

*Multivariable logistic regression restricted to plausible reporters and under-reporters (n = 169, 56 under-reporting events; McFadden pseudo-R² = 0.053). All predictors entered simultaneously. OR, odds ratio; CI, confidence interval. An odds ratio below 1 for dialysis vintage indicates that longer time on dialysis was associated with lower odds of under-reporting.*

**S4 Table. Nutritional and clinical variables by dialysis frequency.**

| **Variable** | **Twice weekly (n = 148)** | **Thrice weekly (n = 33)** | **p** |
| --- | --- | --- | --- |
| Energy (kcal/day) | 1486.6 ± 337.1 | 1424.1 ± 247.4 | 0.229 |
| Protein (g/day) | 56.8 ± 14.8 | 55.9 ± 10.5 | 0.676 |
| DEI (kcal/kg/day) | 23.6 ± 5.5 | 23.3 ± 4.6 | 0.705 |
| DPI (g/kg/day) | 0.90 ± 0.25 | 0.91 ± 0.18 | 0.811 |
| EI:BMR ratio | 0.90 ± 0.25 | 0.86 ± 0.15 | 0.263 |
| BMI (kg/m²) | 22.4 ± 3.8 | 24.6 ± 5.5 | 0.042 |
| Albumin (g/L) | 35.9 ± 4.4 | 37.6 ± 3.6 | 0.023 |
| Potassium (mmol/L) | 4.64 ± 0.96 | 5.02 ± 0.94 | 0.044 |
| Phosphorus intake (mg/day) | 818.4 ± 187.2 | 787.6 ± 141.6 | 0.295 |
| Energy, DEI 30–35 kcal/kg/day | 19/143 (13.3) | 1/33 (3.0) | 0.129 |
| Protein, DPI ≥1.0 g/kg/day | 48/143 (33.6) | 10/33 (30.3) | 0.838 |
| Poor appetite, n (%) | 64 (43.2) | 12 (36.4) | 0.547 |
| Invalid reporting, n (%) | 50 (34.5) | 12 (36.4) | 0.998 |

*Continuous variables are mean ± SD, compared using Welch's t test. Adherence proportions are n/N (%), compared using Fisher's exact test. Appetite and reporting-plausibility distributions were compared using Pearson's chi-squared test. Three participants receiving once-weekly dialysis are grouped with the twice-weekly category.*
