# Supplementary material for: Dietary intake patterns and KDOQI guideline compliance among hemodialysis patients in Bangladesh: Gaps, misreporting, and clinical implications
Source: PLoS One. 2026 Oct 12;21(10):e0360654. doi: 10.1371/journal.pone.0360654 (PMC13657316; doi:10.1371/journal.pone.0360654)
Supplement: Supplementary file 3 [file pone.0360654.s005.docx]

**Response to Reviewers- First Round**

Manuscript PONE-D-26-00308

*Dietary Intake Patterns and KDOQI Guideline Compliance Among Hemodialysis Patients in Bangladesh: Gaps, Misreporting, and Clinical Implications*

Corresponding authors: Shakil Ahmed and Tanjina Rahman

Dear Editors,

Thank you for the opportunity to revise our manuscript, and please convey our thanks to both reviewers. Their comments identified two problems that went beyond presentation: an internal contradiction in how we described the appetite results, and an analysis that leaned on a subgroup of five participants. Addressing these properly required us to return to the primary dataset and recompute every analysis in the paper.

Page and line numbers below refer to the marked-up version (Revised Manuscript with Track Changes), which carries continuous line numbering. Reviewer comments appear in shaded boxes.

# **Reviewer #1**

## **Comment 1:** The over-reporting group included only five participants. Therefore, treating this group as an independent comparison group in repeated ANOVA-based analyses may lead to unstable estimates and overinterpretation. The authors should interpret this group more cautiously or consider supplementary analyses comparing valid versus invalid reporters.

We agree, and this comment reshaped the analysis. Three changes follow from it. First, a pre-specified sensitivity analysis now combines under- and over-reporters into a single invalid-reporter category (n = 62) and compares them with plausible reporters (n = 116). Second, Fisher's exact test replaces the chi-squared test in every categorical comparison involving the five over-reporters, and the test used is stated in each table footnote. Third, the over-reporter column is retained in the descriptive tables for completeness but is explicitly identified as unstable, and no conclusion in the revised manuscript rests upon it.

We are particularly grateful for this suggestion, because following it led directly to the valid-versus-invalid contrast that became the paper's central finding.

**Location in revised manuscript:** Methods, p 6, L 190–196; Results, p 15 (Plausible versus invalid reporters); Table 2 footnote, p 8; Strengths and Limitations, p 20.

## **Comment 2:** There appears to be inconsistency between the results and discussion regarding appetite status. In the results, patients with poor appetite appear to have higher BMI and higher energy intake than those with good appetite, whereas the discussion states that poor appetite was associated with lower energy intake. The authors should revise this interpretation according to the data shown in Table 08.

The reviewer is correct and we apologise for the contradiction. Recomputation confirms that energy intake was lowest in the good-appetite group, and that the significant omnibus difference across appetite categories (F(2, 175) = 3.83, p = 0.024) is driven entirely by the good-versus-fair contrast; in Holm-adjusted pairwise comparisons, good versus poor and fair versus poor were both non-significant (p = 0.258). Protein intake, weight-adjusted intake, BMI and the EI:BMR ratio did not differ across appetite categories at all. Both the Results and the Discussion have been rewritten to state this plainly rather than to assert the conventional pattern.

Checking this also revealed a second, related error in the original submission, which stated that appetite did not differ between valid and invalid reporters. It does: invalid reporters were significantly more likely to rate their appetite at either extreme (χ²(2) = 7.50, p = 0.024). We now report this association and use it to advance a specific interpretation, namely that a poor appetite rating and an implausibly low recorded intake may both reflect the same reporting tendency rather than a true energy deficit. We present this as a hypothesis requiring an objective intake marker to test, not as a conclusion.

**Location in revised manuscript:** Results, p 16, L 358–375; Discussion, p 19, L 418–428; Table 7, p 16–17.

## **Comment 3:** The definition of KDOQI guideline compliance should be clarified. It is unclear whether the reported 13.6% compliance rate refers to energy intake alone, combined energy and nutrient targets, or another composite indicator. The authors should clearly describe how compliance was calculated and which nutrients were included.

A dedicated Methods subsection now defines adherence as the proportion of patients meeting a single, explicitly named target, states that composite adherence is reported separately and labelled as such, and gives the denominator used for every weight-adjusted figure (n = 176 of the 178 in the analytic sample). Every adherence figure in the paper now names its target.

The figure the reviewer queried has been superseded by recomputed values: energy adherence (30–35 kcal/kg/day) is 20/176 (11.4%); protein adherence is 58/176 (33.0%) at the 1.0 g/kg/day threshold and 16/176 (9.1%) at 1.2 g/kg/day; composite adherence is 18/176 (10.2%) and 9/176 (5.1%) respectively.

**Location in revised manuscript:** Methods, p 5, L 143–158 (Reference standards and definition of adherence); Results, p 14, L 303–316; Table 6, p 14–15.

## **Comment 4:** The discussion should better contextualize the comparison with KDOQI recommendations. In this cohort, 80.1% of patients received hemodialysis twice weekly, which differs from settings where thrice-weekly dialysis is standard. Dialysis frequency may influence appetite, uremic symptoms, nutritional intake, and nutritional status.

We agree this needed addressing, and we have done so with data rather than with argument. A new Results subsection and S4 Table compare patients dialysing twice and three times weekly. Contrary to our own expectation, reported energy intake (1485.8 ± 339.4 versus 1424.1 ± 247.4 kcal/day, p = 0.236), protein intake (p = 0.696), weight-adjusted energy (p = 0.722), poor appetite (p = 0.547) and invalid reporting (p = 0.998) did not differ by dialysis frequency. Patients dialysing three times weekly had higher serum albumin (p = 0.021) and higher BMI (p = 0.042).

The Discussion now reports this null result, acknowledges that it conflicts with Nieves-Anaya and colleagues, who found worse nutritional and hydration status among twice-weekly patients in a comparably resourced setting, and offers two explanations for the discrepancy: our small thrice-weekly subgroup (n = 33) and the absence of residual urea clearance data, without which we cannot say whether twice-weekly dialysis delivered adequate total clearance. We also note that the discussion paragraph in our previous version asserted a causal role for the dialysis prescription and cited three references that did not support it; that paragraph has been replaced and the citations corrected.

**Location in revised manuscript:** Results, p 16, L 350–357; S4 Table; Discussion, p 19, L 440–458.

## **Comment 5:** The statistical methods require clarification. Given the very small number of over-reporters, some categorical comparisons may not meet the assumptions of the chi-square test. The authors should consider Fisher's exact test where appropriate. In addition, the issue of multiple comparisons should be briefly addressed.

The Statistical analysis section has been rewritten. It now specifies how distributional assumptions and homogeneity of variance were assessed; reports F with both degrees of freedom and eta squared for every analysis of variance; applies Holm-adjusted pairwise Welch tests where an omnibus test was significant; reports t with Welch-adjusted degrees of freedom, mean differences with 95% confidence intervals and Cohen's d for two-group comparisons; specifies the multivariable logistic regression model in full; and substitutes Fisher's exact test wherever an expected cell count fell below five, with the test used stated in each table footnote. Exact p values are now reported throughout for all values ≥0.001, replacing the 'NS' notation used previously.

On multiple comparisons, we have chosen not to apply a formal correction across the many nutrients examined, and instead state explicitly that nominal p values are reported and that isolated significant findings should be regarded as hypothesis-generating rather than confirmatory. We judged this preferable to a correction that would be arbitrary in a descriptive study of this kind, but we would of course reconsider if the reviewer prefers.

**Location in revised manuscript:** Methods, p 6, L 187–205; footnotes to Tables 1–7.

## **Comment 6:** The manuscript requires careful editing. There are typographical errors, inconsistent terminology, unclear unit expressions, possible discrepancies in sample sizes, and inconsistencies between the text and tables.

The manuscript has been comprehensively edited. 'Bicbonate' is corrected to 'Bicarbonate'; the corrupted cell in the macronutrient table is repaired; hyphenation of 'under-reporter' and 'over-reporter' is standardised; and the two missing word spaces in the Conclusion are fixed. Biochemical results are reported in SI units, with albumin and haemoglobin in g/L, glucose in mmol/L and total iron-binding capacity in µmol/L. Vitamin K is correctly expressed in µg. Tables are now numbered consecutively without the previous gap.

On sample sizes and text–table agreement, we took a structural approach rather than a proof-reading one. Every table is now generated directly from the primary dataset by script, so the tables and the text cannot disagree. This resolves the BMI-category discrepancy, in which categories summed to 175 against a stated denominator of 178; they now sum to 178. Denominators are given for every proportion, and numerators are shown alongside percentages throughout.

**Location in revised manuscript:** Throughout; see in particular Table 1 (p 7–8), Table 3 (p 9–10), Table 4 (p 11) and Results, p 9.

# **Reviewer #2**

## **Introduction, comments 1 and 2:** The introduction states the objective twice, initially in lines 88-90, with a repeat of the same objective in lines 92-99 but with further detail. To give the introduction better structure, the primary objective should be mentioned in detail once in the introduction without any repetition.

The duplication has been removed. The Introduction now closes with a single objectives paragraph that separates one primary aim from three secondary aims. The primary aim has also changed in substance, and now states the question the revised paper actually answers: how screening self-reported dietary intake for plausibility alters estimates of guideline adherence.

**Location in revised manuscript:** Introduction, p 4, L 90–99.

## **Methodology, comments 1–7:** The methodology needs further details: sampling and inclusion/exclusion criteria; sample size calculation/justification; number of centers; how the recruitment process was performed, whether random, convenience-based, or stratified by center; inclusion/exclusion criteria including type of feeding, oral nutritional supplements, current acute illness or hospitalization; and whether dietary recalls were collected by trained dieticians.

All seven points are now addressed. Recruitment was by consecutive convenience sampling from four hospital-based units, one per district, with no stratification or randomisation by centre. Five inclusion and five exclusion criteria are listed and cover feeding route (exclusively oral, with or without oral nutritional supplements; enteral and parenteral feeding excluded), clinical stability, and hospitalisation or acute intercurrent illness in the preceding two weeks. No a priori power calculation was performed; we explain why and report the achieved precision (±7.3 percentage points for a proportion near 50%). Recalls were administered face to face by nutrition graduates trained in the multiple-pass method and supervised by the study nutritionists, using a standardised probing script to reduce interviewer variability.

**Location in revised manuscript:** Methods, p 4–5, L 100–142.

## **Methodology, comment 8:** EI:BMR method was used in the manuscript. However, I believe there needs further justification for the specific cut-offs used and if they are appropriate for hemodialysis patients. Additionally, they should clarify if the post-dialysis or dry weight of patients were used for the EI:BMR calculation.

We now state that the thresholds are the Goldberg cut-offs, explain that they derive from the physiological limits of sustainable energy balance, and cite their prior application in maintenance haemodialysis and CKD cohorts. We confirm explicitly that post-dialysis (dry) body weight was used for all BMR and weight-adjusted calculations, and give the reason: the Goldberg approach assumes weight stability, and interdialytic fluid gain inflates pre-dialysis weight.

We also accept the concern underlying the question. The Goldberg cut-offs have not been formally validated in maintenance haemodialysis, where fluid shifts, altered resting energy expenditure and reliance on predictive BMR equations may all bias classification. Because our principal finding now depends on this screening step, we state this limitation directly rather than in passing, and note that the direction of any residual misclassification is unknown.

**Location in revised manuscript:** Methods, p 5, L 159–172; Strengths and Limitations, p 20.

## **Results, comment 1:** The manuscript mentions the KDOQI targets of 30-35 kcal/kg/day and protein of 1.2g/kg/day. However, the 2020 KDOQI guidelines mention protein intake of 1.0-1.2 g/kg/day. The authors should specify which version of KDOQI was used, and justify the thresholds to avoid 1.2g/kg/day as the only acceptable minimum.

We now specify the 2020 KDOQI update and report adherence against both bounds throughout: 33.0% reach ≥1.0 g/kg/day and 9.1% reach ≥1.2 g/kg/day, with ≥1.0 g/kg/day treated as the minimum acceptable target and ≥1.2 g/kg/day retained for comparability with earlier literature.

This comment prompted us to re-examine our other reference values, and we found a related error the reviewer did not raise. Our micronutrient benchmarks for folate (1000 µg/day) and vitamin B6 (10 mg/day) were the supplementation doses recommended for dialysis patients, not dietary reference intakes. A diet benchmarked against a supplement dose fails by construction, and these have been replaced with the appropriate dietary reference intakes. We are grateful that the comment led us to check.

**Location in revised manuscript:** Methods, p 5, L 143–158; Results, p 14, L 303–316; S1 and S2 Tables.

## **Results, comments 2–4:** The results contain several inconsistencies. Table 2 shows 112 acceptable, 61 under-reporters, and 5 over-reporters, totaling 178, not 181. Lines 168-169 mention acceptable reporting was observed in 55.4% of participants, while 30.2% underreported and 2.5% over reported, yet 112/178 is approximately 62.9%, not 55.4%.

Both errors are confirmed and corrected. Recomputation from the primary dataset gives 116 plausible reporters (65.2%), 57 under-reporters (32.0%) and 5 over-reporters (2.8%), totalling 178. The denominator of 178 rather than 181 reflects three participants whose weight could not be measured, and this is now stated where the figure first appears. These values are consistent across the Abstract, Results, Table 2 and the figures. We are grateful for the arithmetic check.

**Location in revised manuscript:** Results, p 7, L 220–228; Table 2, p 8.

## **Results, comment 5:** The over reporter’s group may be too small to produce any meaningful conclusions. Only five patients were classified as overreporters. Any comparisons involving this group are statistically unstable.

Agreed, and addressed by the same changes described under Reviewer 1, comment 1. No conclusion in the revised manuscript depends on the over-reporter subgroup, and the instability is stated wherever the group appears.

**Location in revised manuscript:** Results, p 15; Strengths and Limitations, p 20.

## **Minor comments:** Few spelling mistakes/language errors: Introduction 'he burden' instead of 'The burden'; Table 1 'Bicbonate' instead of 'Bicarbonate'; 'haemodialysis' and 'hemodialysis' used inconsistently; 'over reported/under reporters' inconsistently hyphenated.

All four are corrected. 'The burden' now reads correctly in the Introduction; 'Bicarbonate' appears correctly in Table 1; spelling has been standardised throughout the manuscript; and 'under-reporter' and 'over-reporter' are consistently hyphenated.

**Location in revised manuscript:** Introduction, p 3; Table 1, p 7, L 217; throughout.

# **Journal requirements**

## **Requirement 1:** PLOS ONE style

The manuscript has been reformatted to PLOS ONE requirements: continuous line numbering and page numbers, double spacing, no more than three heading levels, a short title, SI units, an Acknowledgments section, and Supporting Information captions at the end of the file. Funding and competing interests statements have been removed from the manuscript file and are supplied through the submission system, as the guidelines require.

The reference list has been converted to Vancouver style with NCBI-abbreviated journal titles and up to six authors before 'et al.', and the 'Uncategorized References' heading artefact has been removed. Several entries have been corrected: the KDIGO and National Kidney Foundation corporate authors were malformed; the 2020 KDOQI guideline was cited as its erratum notice rather than the guideline itself; the StatPearls chapter lacked source details; and the Sahathevan reference had a malformed author string and missing volume. We also removed one citation that did not support the statement it was attached to and replaced it with two that do.

**Location in revised manuscript:** Throughout; References, p 22–24.

## **Requirement 2:** Data availability

We have chosen the open option. A minimal anonymised dataset sufficient to reproduce every finding in the paper is supplied as S1 Dataset. It contains no direct identifiers; subject codes, dates of birth and admission dates have been removed and cannot be reconstructed from the file. The Data availability statement has been updated accordingly and we will update the statement in the submission form.

**Location in revised manuscript:** Data availability statement, p 21, L 524–525; S1 Dataset.

## **Requirement 3:** Tables 6 and 7

Both tables are provided in full. Following the reduction in the number of main tables, the vitamin and mineral tables are now supplied as S1 Table and S2 Table, and both have been rebuilt from the primary dataset with test statistics, exact p values, the reference intake applied and the number and proportion of participants meeting it. They are cited in the Results.

**Location in revised manuscript:** Results, p 14, L 284; S1 Table and S2 Table.

## **Requirement 4:** Recommendations to cite specific works

Neither reviewer nor the editor recommended specific publications for citation, so no citations have been added on that basis.

# **Additional changes made on our own initiative**

We think it right to flag changes that neither reviewer requested.

1. Reframing of the paper. Restricting analysis to plausible reporters raises apparent adherence to intake targets (protein from 33.0% to 47.4%, energy from 11.4% to 15.5%) and lowers apparent adherence to restriction targets (sodium from 73.6% to 65.5%, phosphorus from 88.2% to 84.5%). Because the same measurement error exaggerates deficiency and manufactures restriction compliance in the same patient, we now treat this as the paper's principal contribution. A new table and figure present it.

2. Predictors of under-reporting. A multivariable logistic regression (n = 169, 56 events) found dialysis vintage to be the only independent predictor (adjusted odds ratio 0.81 per additional year, 95% CI 0.67–0.96). This does not support the statement in our original submission that under-reporting was more common among women and among patients with higher BMI; neither association survived adjustment, and we have corrected the claim.

3. Limitations. We now acknowledge the absence of Kt/V, normalised protein catabolic rate and a formal malnutrition score, noting that normalised protein catabolic rate in particular would have provided an objective cross-check on reported protein intake. We also state that prescribed renal vitamin supplements were not captured and that residual urea clearance was not measured.

4. Figures. All figures have been rebuilt to PLOS specifications as 300 dpi LZW-compressed TIFF files, with figure titles removed from the image files and placed in the manuscript captions, and with the legend overlap in the previous Fig 1 corrected.

5. Tone. Language in the Conclusion that outran the evidence has been removed, and the conclusions are now confined to what a cross-sectional convenience sample can support.

# **Response to Reviewers - Second Round (Manuscript PONE-D-26-00308R1)**

We thank the Academic Editor and both reviewers for their continued attention to our manuscript. Reviewer #1 has confirmed that all previous comments have been addressed. Reviewer #2 raises one further point of clarification, which we address in full below, together with the two journal requirements accompanying the decision letter.

# **Journal requirements**

## **Requirement 1:** If the reviewer comments include a recommendation to cite specific previously published works, please review and evaluate these publications to determine whether they are relevant and should be cited.

No specific publications were recommended for citation by either reviewer or the Academic Editor in this round, so no citations have been added on this basis.

## **Requirement 2:** Please review your reference list to ensure that it is complete and correct. If you have cited papers that have been retracted, please include the rationale for doing so in the manuscript text, or remove these references and replace them with relevant current references.

We have reviewed the full reference list and confirm that none of the cited works has been retracted.

# **Reviewer #1**

**Reviewer #1:** All comments have been addressed.

We thank the reviewer for confirming this and for their earlier comments, which substantially improved the manuscript.

# **Reviewer #2**

## **Comment 1 (Round 2):** I thank the authors as they have substantially addressed my previous comments. However, further clarification is still required regarding the EI:BMR plausibility assessment. The manuscript states that cut-offs of <0.8, 0.8–1.3 and >1.3 were applied, while the Table 2 footnote states that the Goldberg ratio was adjusted for physical activity level. Please clarify how physical activity level was determined, what PAL values were used, and how this adjustment was incorporated into the calculation.

We thank the reviewer for this comment, which identified a genuine omission in our methods description. We clarify each point in turn.

**How PAL was determined.** A single fixed PAL value was applied to the whole cohort rather than derived from an individual activity questionnaire, as physical activity was not measured directly in this study.

**What value was used.** A PAL of 1.2 was applied, corresponding to the lower bound of physiologically plausible activity for a chair-bound or minimally ambulant lifestyle. This value is supported by doubly-labelled-water data from 574 free-living subjects, in which chair- or bed-bound individuals showed a mean PAL of approximately 1.2 (Black, 2000). We considered this appropriate given the characteristically sedentary lifestyle of maintenance haemodialysis patients, and a directly comparable cut-off has been applied in this same clinical population, used an EI:BMR ratio below 1.2 to identify under-reporting of habitual intake among Malaysian haemodialysis patients (Yusop et al., 2013).

**How the adjustment was incorporated.** Basal metabolic rate (BMR) was estimated for each participant using the sex-specific Harris–Benedict equation, based on post-dialysis (dry) body weight, height and age. This BMR was multiplied by the assumed PAL of 1.2 to estimate each participant’s energy requirement (BMR × PAL). Each participant’s reported energy intake (EI), from the three-day 24-hour dietary recall, was then divided by this PAL-adjusted energy requirement to give the reporting plausibility ratio: EI ÷ (BMR × PAL). This ratio, rather than the unadjusted EI:BMR ratio, was used to classify participants as under-reporters (<0.8), plausible reporters (0.8–1.3), or over-reporters (>1.3), following the Goldberg cut-off framework (Goldberg et al., 1991; Black, 2000), previously applied in maintenance haemodialysis and chronic kidney disease cohorts (Mafra et al., 2012; Fassett et al., 2007).

**Worked example.** For a participant with an estimated BMR of 1356.8 kcal/day and a reported energy intake of 1589.1 kcal/day: energy requirement = 1356.8 × 1.2 = 1628.2 kcal/day; reporting plausibility ratio = 1589.1 ÷ 1628.2 = 0.98, which falls within the 0.8–1.3 band and is classified as a plausible reporter.

We have amended the Methods section (Assessment of reporting plausibility) and the Table 2 footnote to state the PAL value, its derivation, and its incorporation into the calculation explicitly, so that the classification is now fully reproducible from the manuscript text alone. We have also removed the Ocké (2013) reference, which had been cited in this section but does not, on re-examination, support the application of the Goldberg cut-off in dialysis or chronic kidney disease cohorts; the correct supporting citations (Mafra et al., 2012; Fassett et al., 2007) were already present elsewhere in the manuscript and are now cited at this point instead.

**Location in revised manuscript:** Methods, Assessment of reporting plausibility; page number 5, Line number 160-164, yellow colour highlighted.

We hope these revisions address the reviewers' concerns. The comments were detailed and exacting, and the paper is a better one for them.

Yours sincerely,

Shakil Ahmed and Tanjina Rahman, on behalf of all authors
